# Correspondence

**Published:** 1882-10

**Authors:** 


### CORRESPONDENCE.

*Editor Atlanta Medical Register:*

Dear Sir—It has occurred to one of your subscribers that a small portion of your space might be profitably employed by the insertion of an extract from the code of medical ethics and ordinances, of the American Medical Association, as follows:

Article 1st, section 3d, of the code of ethics, states that, "It is derogatory to the dignity of the profession to resort to public advertisements, or private cards, or hand-bills, inviting the attention of individuals affected with particular diseases, publicly offering advice and medicine to the poor gratis, or promising radical cures, or to publish cases and operations in the daily prints, *or suffer such publications to be made,* to invite laymen to be present at operations, to boast of cures and remedies, to adduce certificates of skill and success, *or to perform any other similar acts.* These are the ordinary practices of empirics and are highly reprehensible in a regular physician."

Among the ordinances of the association as explanatory of and supplementary to the foregoing, it is

"Resolved, That this Association recognizes specialties as proper and legitimate fields of practice."

"Resolved, That specialists shall be governed by the same rules of professional etiquette as have been laid down for general practitioners."

"Resolved, That it shall not be proper for specialists publicly to advertise themselves as such, or to assume any title not specially granted by a regularly chartered medical college."

"Resolved, That private hand-bills addressed to members of the medical profession, or by cards in medical journals, *calling the attention of professional brethren to themselves as specialists,* be declared in violation of the code of ethics of the American Medical Association." (Vide Transactions. Vol. XX, P. 28).

Upon these extracts which are designed to regulate the professional conduct of medical gentlemen who profess to be guided by the laws of the American Medical Association, I desire to remark, very briefly, that the history of its transactions, will, I think, bear out fully the assertion, that specialists have never been encouraged, but barely tolerated, and the sentiment of the great mass of regular medical men has been alike opposed to the assumption of special skill upon the part of any member of the profession

The only form of specialism which has been in real accord with *the spirit* of the code of ethics, is that which has originated in long, laborious and eminent experience in some particular department of medical science upon the part of general practitioners and which has involuntarily and unsought, commanded the confidence and recommendation of the profession. That the whole subject is full of difficulty and injury to the profession, I think no candid and intelligent physician will gainsay, and in my judgment it is equally certain that the very best that can be said of specialties in medicine is that they seem to be *inevitable evils.*

What reflecting medical man does not know that the popular idea of necessary superior skill upon the part of those, either in or out of the regular profession, who assume the role of specialists, is utterly fallacious, and that the constant tendency of such practitioners is to a narrow and routine diagnosis and treatment of disease, while the temptation to resort to extraordinary and unprofessional methods to advertise their special superiority to their medical brethren is too strong for many of those who are covered by the code of ethics. Out of this toleration of the practice of specialties upon the part of the American Medical Association, under the unwise pressure of the influence of a number of really eminent men (and from whose chaplet of honor I would not pluck a feather's weight, if in my power), has grown up a mass of pseudo-specialism in the regular profession which is rapidly demoralizing and degrading all forms of special practice. If there is no remedy for this evil, and we are compelled to bear the infliction, let us endeavor to keep up some slight line of demarkation (which is being rapidly obliterated) between legitimate specialism and that without the pale of professional recognition. To this end I invoke the aid of your journal within its sphere, if your views should accord with those of your

OCCASIONAL CORRESPONDENT.

